# Soluble factors from biofilm of *Candida albicans* and *Staphylococcus aureus* promote cell death and inflammatory response

**DOI:** 10.1186/s12866-017-1031-5

**Published:** 2017-06-30

**Authors:** Kassia de Carvalho Dias, Paula Aboud Barbugli, Fernanda de Patto, Virginia Barreto Lordello, Letícia de Aquino Penteado, Alexandra Ivo Medeiros, Carlos Eduardo Vergani

**Affiliations:** 1 Department of Dental Materials and Prosthodontics, Oral Rehabilitation Program–Araraquara School of Dentistry UNESP–Univ. Estadual Paulista, Centro, Araraquara, SP 14801903 Brazil; 2 Department of Biological Sciences, School of Pharmaceutical Sciences, UNESP–Univ Estadual Paulista, Araraquara, São Paulo Brazil

**Keywords:** Biofilm, Cytokines, *Candida albicans*, *Staphylococcus aureus*, Keratinocytes, Macrophages

## Abstract

**Background:**

The objective of this study was to better understand the effects of soluble factors from biofilm of single- and mixed-species *Candida albicans* (*C. albicans)* and methicillin-sensitive *Staphylococcus aureus* (MSSA) cultures after 36 h in culture on keratinocytes (NOK-si and HaCaT) and macrophages (J774A.1). Soluble factors from biofilms of *C. albicans* and MSSA were collected and incubated with keratinocytes and macrophages, which were subsequently evaluated by cell viability assays (MTT). Lactate dehydrogenase (LDH) enzyme release was measured to assess cell membrane damage to keratinocytes. Cells were analysed by brightfield microscopy after 2 and 24 h of exposure to the soluble factors from biofilm. Cell death was detected by labelling apoptotic cells with annexin V and necrotic cells with propidium iodide (PI) and was visualized via fluorescence microscopy. Soluble factors from biofilm were incubated with J774A.1 cells for 24 h; the subsequent production of NO and the cytokines IL-6 and TNF-α was measured by ELISA.

**Results:**

The cell viability assays showed that the soluble factors of single-species *C. albicans* cultures were as toxic as the soluble factors from biofilm of mixed cultures, whereas the soluble factors of MSSA cultures were less toxic than those of *C. albicans* or mixed cultures. The soluble factors from biofilm of mixed cultures were the most toxic to the NOK-si and HaCaT cells, as confirmed by analyses of PI labelling and cell morphology. Soluble factors from biofilm of single-species MSSA and mixed-species cultures induced the production of IL-6, NO and TNF-α by J744A.1 macrophages. The production of IL-6 and NO induced by the soluble factors from biofilm of mixed cultures was lower than that induced by the soluble factors from biofilm of single-species MSSA cultures, whereas the soluble factors from biofilm of *C. albicans* cultures induced only low levels of NO.

**Conclusions:**

Soluble factors from 36-h-old biofilm of *C. albicans* and MSSA cultures promoted cell death and inflammatory responses.

## Background

Biofilm represents a microbial community enclosed in a matrix of extracellular polymeric substances (EPS). Biofilm formation is a form of microbial growth in which interacting sessile cells are anchored to a solid substratum and to each other [1, 2]. Among the opportunistic pathogens in the oral cavity, *Candida albicans* (*C. albicans*) is most frequently isolated in denture bases (64.4%) [3]. Methicillin-sensitive *Staphylococcus aureus* (MSSA) has been recovered from 34.4% of denture users, and the combination of these two microorganisms is found in 8.8% of patients [3]. *C. albicans* and MSSA form a mutual alliance that promotes a positive synergism between the species [4, 5], which has been attributed to increased frequency and severity of infectious diseases [6] such as prosthetic stomatitis, the most common form of oral candidosis, with an overall incidence of 11–65% in users of complete prostheses [7–9]. Moreover, *C. albicans* and MSSA have also been co-isolated from individuals with various pathologies, as well as from the surfaces of various biomaterials such as catheters [10, 11].

These opportunistic pathogens can colonize mucous membranes, invade tissues and cause infection [12]. Their pathogenicity is attributed to several factors, such as the ability to develop biofilms, drug resistance, and the production of toxic metabolites and toxins [13]. *C. albicans* and MSSA biofilms are rich in proteases and phospholipase. When these microorganism biofilms are co-cultured, both phospholipase C (PL-C) and proteases (SAP) can be found [5]. Furthermore, the interaction between MSSA and *C. albicans* promotes a strong inflammatory response in polymicrobial infections and modulates the proteomic profiles of biofilm in co-cultures in vitro. These modulatory effects include the expression of several defined and putative virulent proteins, such as CodY, which regulates nutrient acquisition and toxin production [4].

The ability of microorganisms to induce cell damage is a key factor promoting proinflammatory responses leading to recruitment and activation of immune cells, such as neutrophils and macrophages [14]. Significantly higher levels of systemic and local interleukin-6 (IL-6), tumor necrosis factor alpha **(**TNF-α) and IL-1β have been found during the early stages of co-infection than during single infection, regardless of the morphogenesis of *C. albicans* [15]. The concentrations of IL-6 and IL-8 produced by HaCaT keratinocytes increased when incubated with *C. albicans* filtrates [16]. The pathogenicity of MSSA is due to its repertoire of toxins, exoenzymes, adhesins, and immune-modulating proteins [17]. Compared with monomicrobial peritonitis, polymicrobial peritonitis is associated with increased proinflammatory cytokines such as IL-6, keratinocyte chemoattractant, macrophage inflammatory protein-1α, monocyte chemoattractant protein-1, and granulocyte colony-stimulating factor [11]. Soluble factors from biofilm of MSSA are able to induce the production of IL-1β, IL-6, TNF-α, CXCL-8 and CXCL-1 in human keratinocytes as shown by ELISA [18]. The highest concentrations of IL-6 and TNF-α were detected in human mononuclear cells incubated in the presence of MSSA [19].

Therefore, the study investigated the effects of soluble factors derived from *C. albicans* and MSSA biofilms on epithelial cell death and macrophage inflammatory responses. To identify synergism in the pathogenicity and virulence of these microorganisms, the effects of biofilms from single- and mixed-species cultures were compared.

## Results

### Characterization of single- and mixed-species biofilms

The biofilm of mixed MSSA cultures showed higher log_10_CFU/mL than the biofilm of single cultures, although no such difference was observed between single-species and mixed-species *C. albicans*-derived biofilms (Table 1). The pH of soluble factors from biofilm of *C. albicans* (pH = 7.03), MSSA (pH = 6.89), and mixed cultures (pH = 7.00) remained near physiologic levels (pH = 7.0), showing that pH did not affect the cell viability rates.

**Table 1-:** Growth of single and mixed *C. albicans* and MSSA biofilms after 36 h, performed three times in triplicate (*n* = 9 samples)

	Log_10_ CFU/mL		IC (95%)				
Group	Mean	SD	LL	UL	Minimum	Maximum	*p* ^*^
Ca^a^	5.75	0.18	5.61	5.89	5.46	5.99	0.01
MSSA^b^	7.85	0.03	7.82	7.88	7.78	7.91	0.01
Mixed (Ca)^a^	5.84	0.06	5.79	5.90	5.75	5.95	0.50
Mixed (MSSA)^c^	8.39	0.18	8.25	8.53	8.04	8.68	0.01

ANOVA. Tukey post-test = equal letters represent results with no significant difference; different letters represent results with a significant difference

**Fig. 1:**
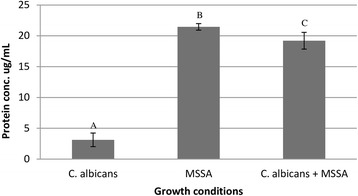
Total protein contained in the biofilm metabolites produced by single and mixed-species biofilms by the Bradford method. Different letters means significant differences (*p*˂0.05)

Protein content measurements by Bradford assay showed a lower total protein level of soluble factors in biofilm from *C. albicans* cultures (3.12 μg/mL ± 1.10) than from MSSA (21.44 μg/mL ± 0.53) or mixed cultures (19.21 μg/mL ± 1.35) (Fig. 1).

### Cell viability

**Fig. 2:**
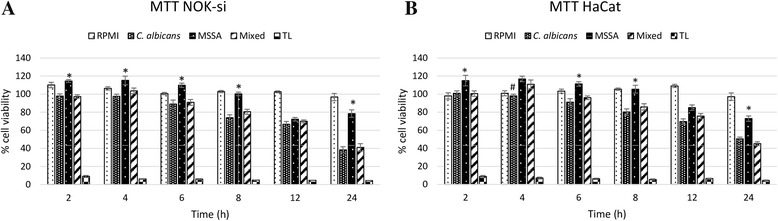
NOK-si (**a**) and HaCat (**b**) cell viability after being in contact with soluble factors from biofilm for 2, 4, 6, 8, 12 and 24 h in three independent experiments, in triplicate for each experimental condition (*n* = 9). *Error bars* represent standard error. * versus *C. albicans* and mixed biofilm. # versus MSSA and mixed biofilm. Analysis by ANOVA, Tukey post-test, *p* ≥ 0.05. RPMI = Negative control, *C. albicans* = soluble factors from biofilm of *C. albicans*, MSSA = Soluble factors from biofilm of MSSA, Mixed = soluble factors from mixed biofilm, TL = lysis buffer

The cell viability of NOK-si cells started to decrease after 6 h of incubation with soluble factors from biofilm of single- *C. albicans* and mixed-species cultures. Cell viability after incubation with MSSA soluble factors remained unaltered, with minimum decrease after 12 h of incubation. Similar results were obtained when HaCaT cells were incubated with soluble factors from biofilm of single- *C. albicans* and mixed-species cultures. The greatest reductions in cell viability were achieved within 24 h for all groups (Fig. 2a and b).

### Cell membrane damage

**Fig. 3:**
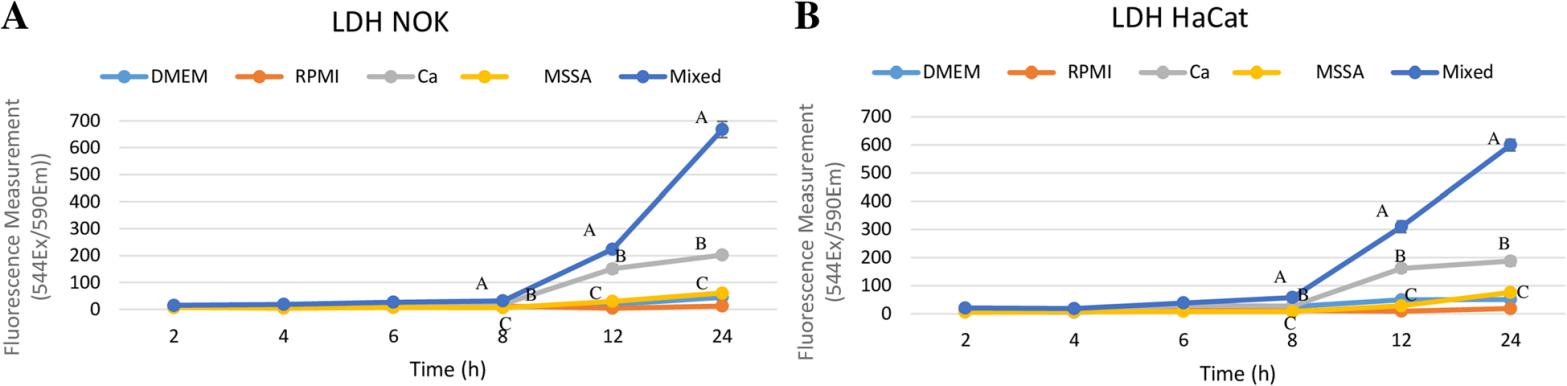
LDH release from NOK-si (**a**) and HaCat (**b**) cells after incubation with soluble factors from biofilm of for 2, 4, 6, 8, 12 and 24 h. Three independent experiments were performed in triplicate for each experimental condition (*n* = 9). DMEM = negative control; RPMI = negative control; *C. albicans* = soluble factors from biofilm of *C. albicans*; MSSA = Soluble factors from biofilm of MSSA; Mixed = soluble factors from mixed biofilm. (Tukey post-hoc test at ρ >0.05). Different letters for results with significant difference in relation to growth conditions (*C. albicans*, MSSA and Mixed biofilm)

The soluble factors from biofilms of mixed cultures significantly increased LDH release compared with those from single-species *C. albicans* and MSSA cultures after 8 h of incubation, reaching higher LDH levelsin 24 h. The soluble factors in biofilm from MSSA induced less LDH release (Fig. 3a and b).

### Cell morphology of keratinocytes

**Fig. 4:**
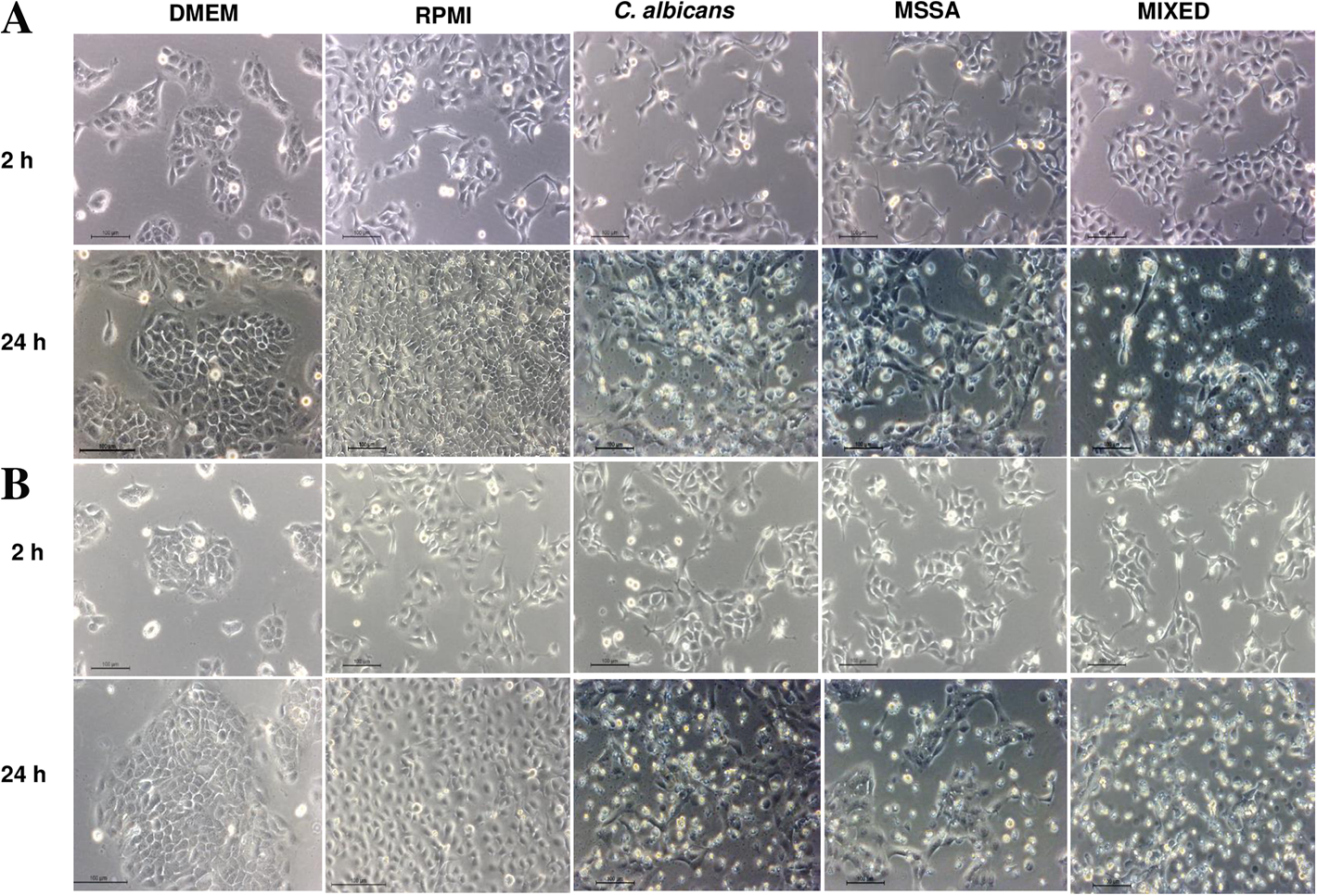
**a** Images obtained from inverted microscope for the control group (DMEM and RPMI) and experimental groups (*C. albicans*, MSSA and Mixed) after 2 and 24 h of contact with NOK-si. **b** Images obtained from inverted microscope for the control group (DMEM and RPMI) and experimental groups (*C. albicans*, MSSA and Mixed) after 2 and 24 h of contact with HaCat. The bar in the images corresponds to 100 μm

No changes in the morphology of NOK-si and HaCaT cells were observed after only 2 h of contact with soluble factors from biofilm of single-species *C. albicans* or MSSA cultures or mixed-species cultures. Major changes in morphology (i.e., loose and spherical cells, cellular debris, and decreased number of cells) were evident after 24 h of incubation with the soluble factors from biofilms of the three types of cultures (Fig. 4a and b).

### Cell death by necrosis or apoptosis

**Fig. 5:**
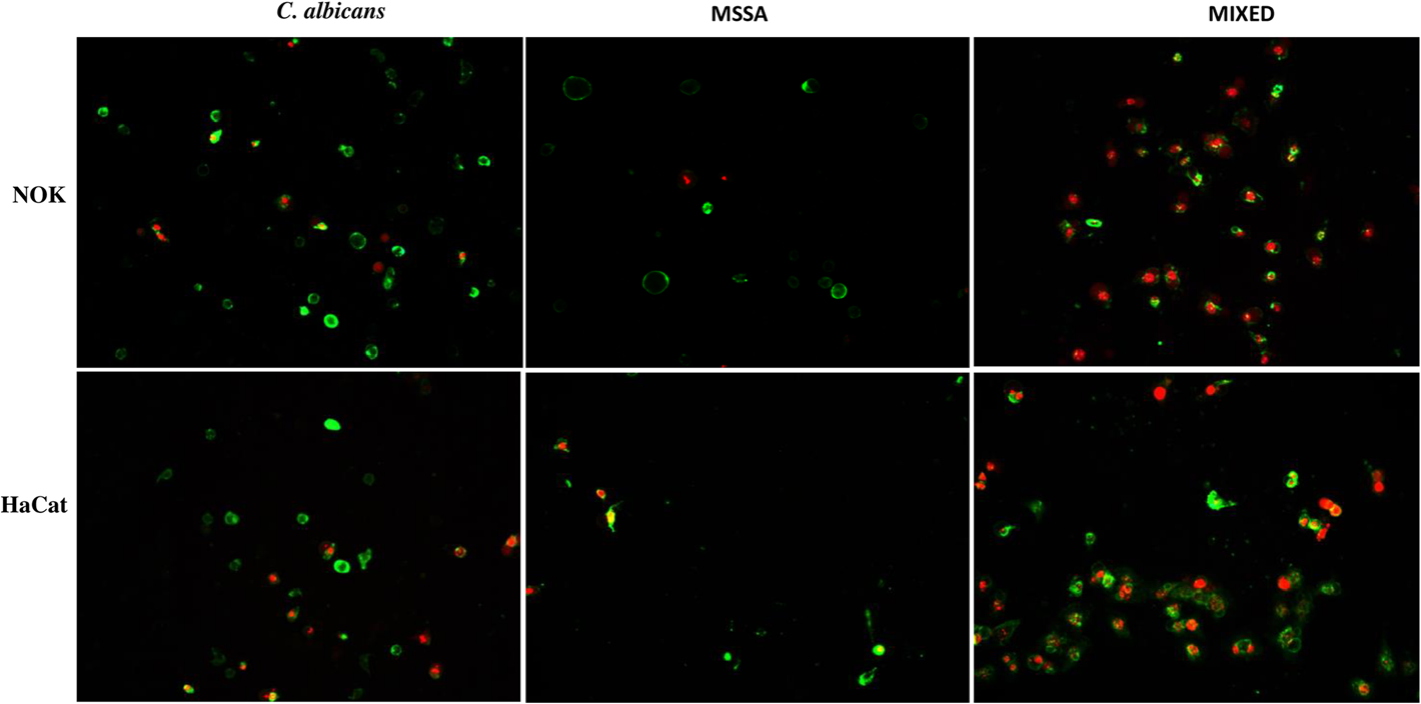
Detection of apoptotic cells (staining positively for annexin V in green) and necrotic cells (staining positively for PI in red) with soluble factors from *C. albicans*, MSSA and mixed biofilm. Magnification ×200

Because we detected more LDH release from the NOK-si and HaCaT cells (which suggested necrosis) after incubation with the soluble factors from biofilm of mixed-species cultures than after incubation with the soluble factors from biofilm of single-species cultures, NOK-si and HaCaT cells were labelled with annexin V and PI. The cells that were incubetedwith the soluble factors from biofilm of mixed cultures were positively labelled with annexin V. However, more abundant PI staining of cells incubated with soluble factors from biofilm of mixed cultures than single-species cultures suggested that the microorganism synergism caused greater disruption in the integrity of epithelial cell membranes, resulting in cell death via late apoptosis or necrosis. Cells were also positively labelled with annexin V and, to a lesser extent, PI when exposed to the soluble factors from biofilm of single-species *C. albicans* cultures. Fewer cells positively labelled with annexin V and PI were observed when the cells were exposed to the soluble factors from biofilm of MSSA cultures (Fig. 5).

### Production of cytokines by macrophages

**Fig. 6:**
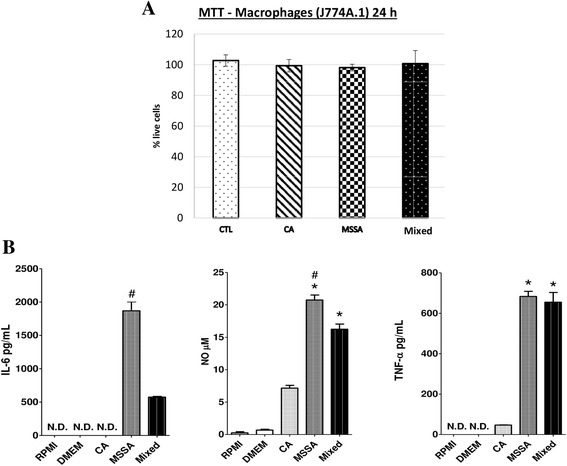
Macrophage response after challenge with soluble-factors produced by *C. albicans* and MSSA single- and mixed-species biofilms. **a** Toxicity percentage according to soluble factors from biofilm in contact with the J774A.1 macrophage, tested on three occasions performed in triplicate (*n* = 9 samples). **b** IL-6 (A), NO (B) and TNF-α (C) production from J774A.1 macrophages after 24 h stimulation with soluble factors from MSSA, *C. albicans* and mixed biofilm. *Error bars* represent standard deviation. Symbols represent statistical differences. IL-6, # versus Mixed; NO e, * versus *C. albicans*; # versus Mixed; TNF-alpha, * versus *C. albicans.* Analysis by ANOVA, Tukey post-test, *p* ≥ 0.05. CLT = control (DMEM), *C. albicans* = *C. albicans* soluble-factors, MSSA = MSSA soluble-factors, Mixed = mixed soluble-factors. N.D. = not detectable

Macrophages exposure to soluble factors from biofilms demonstrated unaltered cell viability (Fig. 6a). ELISA revealed that the J774A.1 macrophages stimulated with soluble factors from biofilms of MSSA cultures had greater production levels of NO (nitric oxide), IL-6 and TNF-α than those exposed to soluble factors from biofilms of *C. albicans* cultures. When stimulated with soluble factors from biofilms of mixed cultures, TNF-α production by J774A.1 cells remained at similarly high levels to those stimulated with soluble factors from biofilms of MSSA cultures*,* with the exception of IL-6 and NO, which were at higher levels in cells stimulated with soluble factors from biofilms of MSSA cultures (Fig. 6b).

## Discussion

The present study demonstrated that soluble factors from biofilm of mixed-species cultures were more pathogenic to epithelial cells than those from biofilms of single-species cultures. Moreover, soluble factors from biofilm of single-species *C. albicans* cultures promoted modest cytokine production by J774A.1 macrophages, whereas soluble factors from biofilm of single MSSA cultures yielded higher levels of cytokines.

Biofilm characterization revealed higher log_10_CFU/mL MSSA in biofilms of mixed-species cultures than in those of single-species cultures (Table 1). Prostaglandin E2 produced by *C. albicans* and present in biofilms of mixed-species cultures consisting of *C. albicans* and MSSA, is known to stimulate the growth of MSSA [20]. Moreover, MSSA has an affinity for *C. albicans* hyphae [4, 5] and accordingly, more MSSA biomass is retained after washing, which may account for the higher log_10_CFU/mL MSSA in biofilms from mixed–species cultures. Additionally, the biofilm of single-species MSSA cultures and the biofilm of mixed-species cultures induced higher levels of total protein production than the biofilm of single-species *C. albicans* cultures (Fig. 1). Although no differences of total protein content were observed between the soluble factors from biofilm of MSSA cultures and mixed cultures, differential in-gel electrophoresis has shown significant differences in the production of 27 proteins by MSSA and *C. albicans* during co-culture biofilm growth, including CodY, which regulates toxin production and nutrient acquisition [4].

The soluble factors from biofilms of mixed-species cultures were more damaging to both NOK-si and HaCaT cells than the soluble factors from biofilms of single *C. albicans* and MSSA cultures. This observation, however, was not evident from the MTT assay, wherein the decreased rates of cell viability were similar between soluble factors from biofilm of single *C. albicans* and mixed cultures. Nevertheless, the LDH assay revealed greater LDH release from both cell types after incubation with the soluble factors from biofilm of mixed cultures. Hydrolytic enzymes, such as phospholipases (PL-C) and proteinase, are metabolites known to be present in *C. albicans* biofilms [21, 22]. Moreover, greater proteinase production has been reported in biofilms of single-species MSSA cultures, whereas biofilms of single-species *C. albicans* cultures presented greater levels of PL-C. When the two microorganisms were co-cultured, both enzymes were produced [5]. The activation of phosphatidylinositol-specific PL-C increases cellular calcium levels and may in turn participate in apoptosis [23]. Thus, PL-C production may be associated with a greater number of apoptotic keratinocyte cells following exposure to soluble factors from biofilms of single-species *C. albicans* and mixed-species cultures (Fig. 5). However, future studies using inhibitors of these enzymes in mixed cultures and monocultures would be required to confirm these hypotheses.

The LDH results demonstrated that soluble factors from 36-h-old biofilms were harmful to NOK-si and HaCaT cells, regardless of the source of the soluble factors. However, soluble factors from biofilm of mixed-species cultures produced more cell membrane damage, indicating the increased pathogenicity of the soluble factors from biofilm of mixed-species cultures (Fig. 3a and b). This more pathogenic behaviour was also demonstrated in the annexin V/PI assays. Under such conditions, the NOK-si and HaCaT cells were double-stained with annexin V and PI. In contrast, when the cells were exposed to soluble factors from biofilm of single-species cultures, few cells were positively stained with PI, although positive PI labelling was more frequent in the *C. albicans* group (Fig. 5). Additionally, *C. albicans* secretes farnesol [24], which is capable to induce apoptosis via activation of caspase, the production of reactive oxygen species (ROS) and the disruption of mitochondrial integrity, resulting in cell death [10, 25, 26].

Interestingly, higher levels of IL-6 production by J774A.1 cells were induced in the presence of soluble factors from biofilm of MSSA cultures. Lower levels of this cytokine were also detected when macrophages were exposed to soluble factors from biofilm of mixed cultures. MSSA toxins (e.g. exotoxins, protein A and α-toxins) do not possess direct cell damaging action, but have a potent effect on cells of the immune system by inducing the overproduction of cytokines [27]. Previous studies have shown that lipoteichoic acid is a potent stimulus that induces IL-6 production by monocyte-like cell lines [28, 29]. IL-6 can inhibit apoptosis during the inflammatory process, keeping cells alive even in highly toxic environments [30]. Through an uncertain mechanism, the soluble factors from biofilm of *C. albicans* interacted with those of *S. aureus*, causing the soluble factors from biofilm of mixed cultures to induce lower levels of IL-6 production by macrophages. However, high levels of TNF-α can trigger the extrinsic apoptotic pathway through recruitment TNF receptor 1 (TNFR1) along with TRADD and other molecules [31]. Furthermore, post-translational modifications can promote RIP1 and TRADD dissociation, leading to the exposure of their death domains (DD), which bind to FADD and recruit caspases-8 and -10, resulting in apoptosis [31]. While we did not perform experiments to determine whether supernatant from macrophages could affect cell death in NOK-si and HaCaT cells, our results suggest that the different amounts of TNF- α and IL-6 generated by macrophages in this microenvironment could affect the cell death of keratinocytes.

The presence of *C. albicans* has been shown to be capable of blocking nitric oxide (NO) production by macrophages [32]. In this study, the soluble factors from biofilm of single-species *C. albicans* cultures likely mediated the same effect. The same behaviour was observed for NO production by J774A.1 cells after exposure to soluble factors from biofilm for 24 h. NO production was observed when the macrophages were incubated with soluble-factors from biofilm of single-species MSSA cultures, and less NO production was observed when the macrophages were incubated with soluble-factors from biofilm of mixed-species cultures than single-species MSSA cultures. In contrast, soluble factors from single-species *C. albicans* cultures induced low levels of NO production by J774A.1 cells (Fig. 6b).

The present study showed that *C. albicans* interacted with *S. aureus* in the microenvironment of 36-h-old biofilm of mixed-species cultures such that the soluble factors of this biofilm were less able to induce IL-6 and NO production than the soluble factors from biofilm of 36-h-old *S. aureus* cultures. This behaviour was not observed for TNF production, which may be due to the lower production of NO induced by the soluble factors from biofilm of mixed-species cultures. Indeed, the induction of NO production has been related to higher levels of TNF-α [33].

This field requires further study to determine the biochemistry of these soluble factors, the mechanisms involved in human cell death following exposure to soluble factors from biofilm of single- and mixed-species cultures, and the inflammatory pathways induced by these factors.

## Conclusion

The results of the present study show that soluble factors from 36-h-old biofilms of *C. albicans* and *S. aureus* cultures promote cell death and inflammatory responses. During the growth of the biofilms, the presence of *C. albicans* evidently enhanced the damage to keratinocytes caused by soluble factors from biofilm of mixed cultures, triggering major necrotic cell death. However, the soluble factors from biofilm of mixed cultures were less capable of inducing the production of proinflammatory cytokines than the soluble factors from biofilm of *S. aureus* cultures.

## Methods

### Microbial strains and growth conditions

*C. albicans* SC5314 and MSSA ATCC25923 microorganisms were used to produce single and dual species biofilms, in accordance with the methodology described by Zago et al. [5]. After incubation, seven freshly grown colonies of MSSA were transferred to 10 mL of TSB medium for pre-inoculum growth at 37 °C for 18 h. For *C. albicans*, 10 freshly grown colonies were transferred to 10 mL of Yeast Nitrogen Base broth culture media (YNB; Difco, Becton Dickinson, Sparks, MD, USA) supplemented with 100 mM glucose for pre-inoculum growth at 37 °C for 16 h. Thereafter, the dilution of the inoculum was performed, and cultures were incubated until the mid-exponential growth phase. The cells of the resultant cultures were harvested and washed twice with sterile phosphate buffered saline solution (PBS, pH 7.2). Microorganisms were resuspended in RPMI-1640 culture medium (Sigma-Aldrich, St. Louis, MO, USA) supplemented with HEPES (25 mM), L-glutamine (2.0 mM) and sodium bicarbonate (2.0 g/L) (Sigma-Aldrich, St. Louis, MO, USA) [34]. The optical densities of the suspensions were standardized to 1 × 10^7^ CFU/mL for both microorganisms.

### Adhesion and biofilm formation

Biofilm formation was carried out in 24-well microplates (TPP Techno Plastic Products AG, Switzerland) [35]. Adhesion and biofilm formation was realized according to protocol recommended by Zago et al. [5]. Experiments were performed in three replicates and repeated in three independent assays. pH was measured using a benchtop pH meter (QX 1500 Plus-Qualxtron, São Paulo, Brazil).

To obtain the soluble factors from the 36-h-old biofilms, media (supernatants) and the microorganisms attached to the wells were removed and filtered through a 0.22-μm low-protein-binding filter (SFCA, Corning, Germany). For epithelial cells (NOK-si and HaCaT), the undiluted soluble factors from biofilm were applied for 2, 4, 6, 8, 12 and 24 h.

### Keratinocyte cell cultures

NOK-si was kindly provided by Prof. Dr. Carlos Rossa Junior (Department of Periodontology, School of Dentistry of Araraquara-UNESP, Brazil) [36], and HaCaT (BCRJ 0341) and J774A.1 macrophages (BCRJ 0121) were purchased from the Rio de Janeiro Cell Bank (BCRJ, RJ, Brazil). The cells were cultured in Dulbecco’s Modified Eagles Medium (DMEM, Gibco BRL, Grand Island, NJ, USA) containing 10% fetal bovine serum (FBS, GIBCO, Grand Island, NY), 100 IU/mL penicillin, 100 mg/mL streptomycin (Sigma Chemical Co., St. Louis, MO, USA) and 2 mL/L glutamine (GIBCO, Grand Island, NY). The cells were maintained at 37 °C under 5% CO_2_ and 80% humidity. The cells were grown until confluence (90%), counted in a Neubauer chamber (magnification ×10) and plated (4.5 × 10^4^ cells/well for NOK-si and HaCaT and 1.0 × 10^5^ cells/well for J774A.1). Cells were used between the 3rd and 8th passages.

### Proteins contained in the supernatant

The total level of soluble protein contained in the soluble factors from biofilm was measured by Bradford protein assay [37], using bovine serum albumin (BSA, Sigma-Aldrich, St. Louis, MO, USA) as the standard. Spectrophotometric measurements were performed at 595 nm (400 EZ Reader; Biochrom, Cambridge, UK).

### Cell viability

For cellular metabolism analyses, the mitochondrial activity of the keratinocytes was measured using the MTT assay [3-(4.5-dimethylthiazole-2-yl) 2.5-diphenyl tetrazolium bromide] (Sigma-Aldrich, St. Louis, MO, USA) [38], which was performed at 2, 4, 6, 8, 12 and 24 h after incubation at 37 °C in 5% CO_2_ in RPMI (negative control), *C. albicans*, MSSA and mixed soluble factors from biofilm, and lysis buffer containing Triton X-100 (LB, positive control). After each period of contact, the cells were washed with PBS. Then, 250 μL of MTT (2.5 mg/mL) were added to each well and the plates were incubated for 4 h. Next, MTT was removed and the formed formazan crystals were solubilized in 250 μL of 2-propanol. Spectrophotometric measurements were performed at 562 nm (Reader 400 EZ; Biochrom, Cambridge, UK).

### Cell membrane damage

The release of the enzyme lactate dehydrogenase (LDH) was determined after 2, 4, 6, 8, 12 and 24 h of contact with the soluble factors using the CytoTox-96 nonradioactive cytotoxicity assay (Promega, Madison, WI) according to the manufacturer’s recommendations. Initially, 100 μL of each control (RPMI, DMEM and lysis buffer containing TritonX-100) and the experimental samples (soluble factors from biofilm) were added in triplicate to a 96-well plate (Thermo Scientific; #31125). Next, 100 μL of the CytoTox-ONE TM reagent was added, and the plate was incubated for 10 min. Subsequently, 50 μL of a “stop” solution were added to each well, and fluorescence was then measured with a filter combination of 544 nm/590 nm (Fluoroskan, FL Ascent, Labsystems, Helsinki, Finland). Culture medium and soluble factors from biofilm were used as blanks.

### Evaluation of keratinocyte morphology

After 2 and 24 h of contact with soluble factors from biofilm, keratinocytes were analysed and photographed by brightfield microscopy using a Leica DMI 3000B microscope (Leica Microsystems, Wetzlar, Germany) soluble factors from biofilm.

### Cell death assay

The type of NOK-si and HaCaT cell death (apoptosis/necrosis) induced by soluble factors from biofilm of MSSA, *C. albicans* and mixed cultures was investigated using annexin V/Alexa Fluor 488 and PI (594 nm) (Molecular Probes, Invitrogen). Annexin V binds specifically to phosphatidylserine residues on cell membranes during apoptosis. PI intercalates with the broken DNA, a typical process of cell necrosis [39, 40]. After 8 h (in the range of 8–12 h, approximately 10 h) of contact with the soluble factors from biofilm of single- and mixed-species cultures, the cells were washed twice with binding buffer (10 mM HEPES/NaOH, 140 mM NaCl, 2.5 mM CaCl_2_, pH = 7.4) and incubated with annexin V (10 μL/well) and 2 μL of PI (100 μg/mL) for 20 min at room temperature. The cells were then washed with PBS and maintained in 10% DMEM for analysis with an inverted fluorescence microscope (Leica DMI 3000B; Leica Microsystems, Wetzlar, Germany).

### Cytokines produced by macrophages

J774A.1 macrophages were continuously exposed to soluble factors from biofilm of *C. albicans*, MSSA or mixed cultures for 24 h, at a ratio of 1:4 (soluble factors from biofilm: cell culture medium) to avoid cell death (as determined experimentally by MTT assays; Fig. 6a). Subsequently, macrophage supernatants were collected, and cytokine (IL-6, TNF-α and NO) production was measured by enzyme-linked immunosorbent assay (ELISA; BD Biosciences, San Jose, CA).

### Statistical analysis

The normality and homogeneity of variances were evaluated using the Shapiro-Wilk and Levene tests. The results were evaluated using one-way analysis of variance (ANOVA) followed by Tukey’s test. A 5% significance level was adopted for all tests performed (*p*˂0.05). All studies were performed in triplicate for each experimental condition and repeated on three independent occasions (*n* = 9).

## Abbreviations

BHI: Brain heart infusion
BSA: Bovine serum albumin
*C. albicans*: *Candida albicans*
CFUs: Colony-forming units
DMEM: Dulbecco’s modified eagle’s
ELISA: Enzyme-linked immunosorbent assay
EPS: Extracellular polymeric substances
FBS: Fetal bovine serum
HaCat: Human adult skin keratinocytes propagated under low Ca2+ conditions and elevated temperature
IL-6: Interleucin-6
J774A.1: Macrophages
LDH: Lactate dehydrogenase
MSSA: Methicillin-sensitive *Staphylococcus aureus*
MTT: 3-(4,5-Dimethylthiazol-2-yl)-2,5-diphenyltetrazolium bromide
NO: Nitric oxide
NOK-si: Spontaneously immortalized normal oral keratinocytes
OD: Optical density
PBS: Phosphate buffered saline
PI: Propidium iodide
PL-C: Phospholipase C
RPMI: Roswell Park Memorial Institute
SAP: Proteases
SDA: Sabouraud dextrose agar
TNF-α: Tumour necrosis factor alpha
TSB: Tryptic soy broth medium
YEPD: Yeast peptone glucose medium
